# Repetitive transcranial magnetic stimulation (rTMS) as a therapeutic option in paraneoplastic cerebellar ataxia – a case report

**DOI:** 10.25122/jml-2022-0156

**Published:** 2022-06

**Authors:** Nicoleta Jemna, Ana Calina Zdrenghea, Georgiana Frunza, Anca Demea, Dafin Fior Muresanu

**Affiliations:** 1 RoNeuro Institute for Neurological Research and Diagnostic, Cluj-Napoca, Romania; 2 Department of Neurosciences, Iuliu Hatieganu University of Medicine and Pharmacy, Cluj-Napoca, Romania

**Keywords:** paraneoplastic cerebellar ataxia, rTMS (repetitive transcranial magnetic stimulation), onco-neuronal antibodies, CBI – cerebellar-brain inhibition, CTC – cerebello-thalamo-cortical, DLPFC – dorsolateral prefrontal cortex, ET – eye-tracking, SKG – statokinesigram, MT – motor threshold, PCA – paraneoplastic cerebellar ataxia, PNS – paraneoplastic neurological syndrome, rTMS – repetitive transcranial magnetic stimulation

## Abstract

Paraneoplastic cerebellar ataxia is a paraneoplastic neurological syndrome (PNS) that can be the first clinical manifestation of underlying cancer. It is usually associated with onco-neuronal antibodies and has no other specific paraclinical feature. After the surgical and oncologic treatment of the primary cancer, the remaining neurological symptoms have limited therapeutic options. We describe a case of severe ataxia as the primary manifestation of ovarian cancer, with a significant clinical and paraclinical improvement of the neurological symptoms after 20 sessions of rTMS intervention.

## INTRODUCTION

Paraneoplastic neurological syndrome (PNS) is a rare finding (1% of oncologic patients) of neurological symptoms induced by a malignancy not caused by direct invasion of cancer or metastasis in the nervous system and not an adverse reaction to the oncological treatment [1]. It is usually associated with onco-neuronal antibodies and may be the first clinical sign of underlying cancer. PNS can affect any part of the nervous system, one of the possible presentations being paraneoplastic cerebellar ataxia (PCA) [2].

rTMS is a non-invasive procedure that can stimulate different brain regions using a magnetic coil applied on the scalp. rTMS is a widely used technique with an expanding range of indications. For example, different rTMS protocols have been used for ataxia, with promising results [3], the majority of them using inhibitory frequencies applied in the posterior areas [4]. Even though there is no guideline recommendation for using rTMS in different etiologies of ataxia, there is evidence to showcase the efficacy and safety [5].

## CASE REPORT

### Patient information

**Figure 1:**
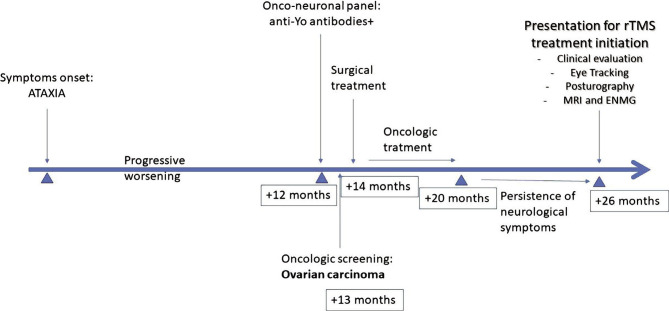
History of the symptoms, diagnostic measures, and treatment administration timeline.

We present the case of a 46 years-old woman with a family history of reproductive system neoplasms and personal medical history of arterial hypertension, who presented to the neurologist for gait and balance disturbances that began 2 years ago with progressive worsening. During the first year of her symptoms, the patient visited multiple healthcare specialists (ear-nose-throat specialist, neurologist) without obtaining a diagnosis, although multiple paraclinical investigations were made (cerebral MRI, electroneuromyography (ENMG) – without pathological findings). After one year, the patient was evaluated through an onco-neuronal panel (antibodies against Hu, Yo, Ri, CRMP5, amphiphysin, Ma2, Tr, recoverin, SOX1) that showed positive anti-Yo antibodies, establishing the diagnosis of PCA. Thus, a neoplastic screening was performed, showing an ovarian carcinoma. The tumor was surgically removed, and the patient underwent adjuvant chemotherapy with Carboplatin and Paclitaxel. Despite the oncologic treatment, the patient's neurological symptoms persisted, having a significant impact on her quality of life (Figure 1).

### Clinical findings

At the time of the presentation, the neurological exam showed horizontal nystagmus, dysarthria, posture, and action tremor – more significant on the right side – inferior limbs dysmetria, ataxic gait – with a wide base, unstable, only possible with unilateral support, difficulties in turning, starting and stopping, globally pendular osteo-tendinous reflexes, hand and feet paresthesia.

At the psychological evaluation, the patient had moderate to severe depression scores, with a score of 28 points in the Beck Depression Inventory-II, and no cognitive dysfunction.

### Diagnostic Assessment

At the presentation in our clinic, we performed a series of investigations. A cerebral MRI showed mild cerebellar atrophy without cortical atrophy and no other abnormalities, while an ENMG examination showed normal transmission speed and amplitude, with no signs of neuropathy.

We performed eye-tracking (ET), a method used to register eye movements to find the direction of a person's gaze, widely used in the last years to diagnose and describe neurological diseases [6]. The ET analysis suggested a disruption in the function of the cerebellar component in regulating the oculomotor circuit.

The posturography examination showed a global antero-posterior body sway score of 56 and a statokinesigram (SKG) area of 451.68 [7], the diagnosis of central nervous system integration deficit being established.

### Therapeutic intervention

Because of the persistence of the patient's neurological symptoms, we decided to perform rTMS for the modulation of the cerebellar function combined with the rTMS-approved protocol for depression. A wide range of rTMS protocols have been used before for ataxia of different etiologies, as reported in the literature, and although no meta-analysis has been performed, the intervention showed promising results.

Using a circular coil, daily sessions were performed for 20 working days. The stimulation site was the cerebellum in each of the following 3 areas: 4 cm to the right of the inion, on the inion, 4 cm to the left of the inion, with 1 Hz frequency and an intensity of 100% of the motor threshold (MT).

Two trains of 10 pulses each were applied, for a total of 60 pulses. At the end of the cerebellar stimulation, we also performed the guideline-approved depression stimulation protocol (10 Hz, left DLPFC – the dorsolateral prefrontal cortex, 3000 pulses, 120% MT).

### Follow-up and outcomes

After rTMS therapy, clinical evaluation, psychological scales, ET, and posturography parameters were significantly improved.

**Figure 2:**
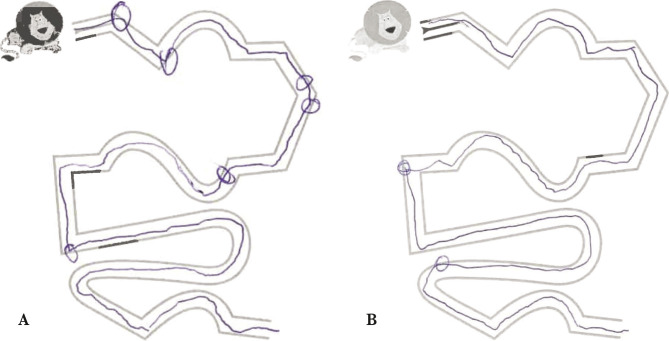
Tracing abilities: A – Before rTMS therapy (4 points of contact); B – After rTMS therapy (2 points of contact).

Clinically, the patient was able to walk without aid, presented improvement in speech, with milder dysarthria and a diminished intensity of the action tremor and dysmetria, and improved tracing abilities (Figure 2 A and Figure 2 B).

The Beck Depression Inventory-II score also showed improvement, with a decrease from 28 to 20 points after rTMS, the psychological assessment showing borderline clinical depression.

**Figure 3:**
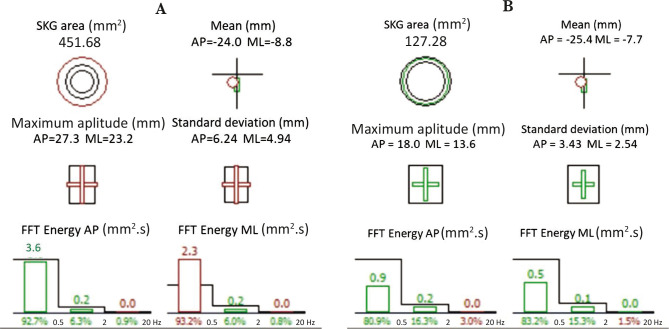
Posturography parameters: A – Before rTMS treatment; B – After rTMS treatment.

**Figure 4:**
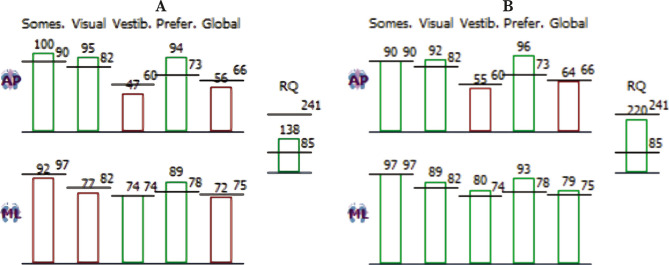
Complete static sensory organisation test – evaluation parameters: A – Before rTMS treatment; B – After rTMS treatment.

Posturography parameters (Figure 3 A, B and Figure 4 A, B) showed improvement of the cerebellar function with an increase in the global anterio-posterior body sway score from 56 to 64 after rTMS, and the statokinesigram (SKG) area decreased from 451.68 mm to 127.28 mm [7].

**Figure 5:**
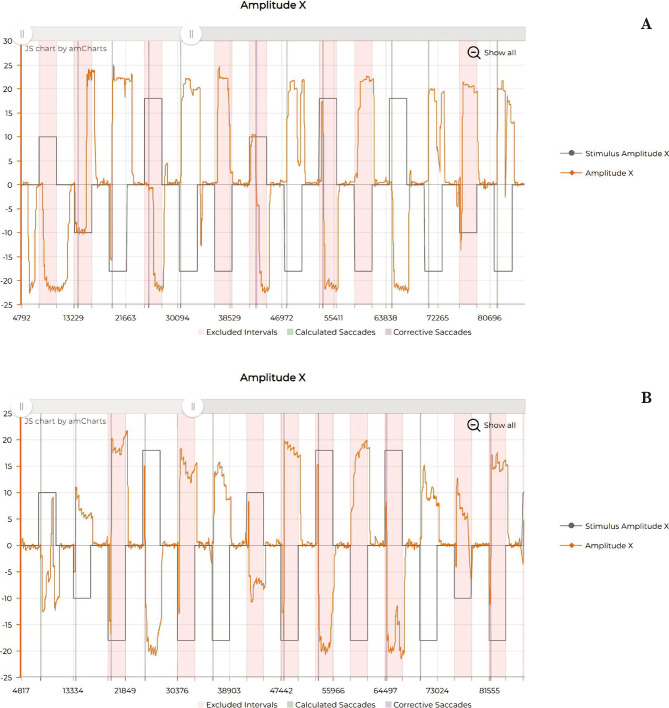
Eye tracking – Antisaccades. A – Before rTMS treatment; B – After rTMS treatment.

**Figure 6:**
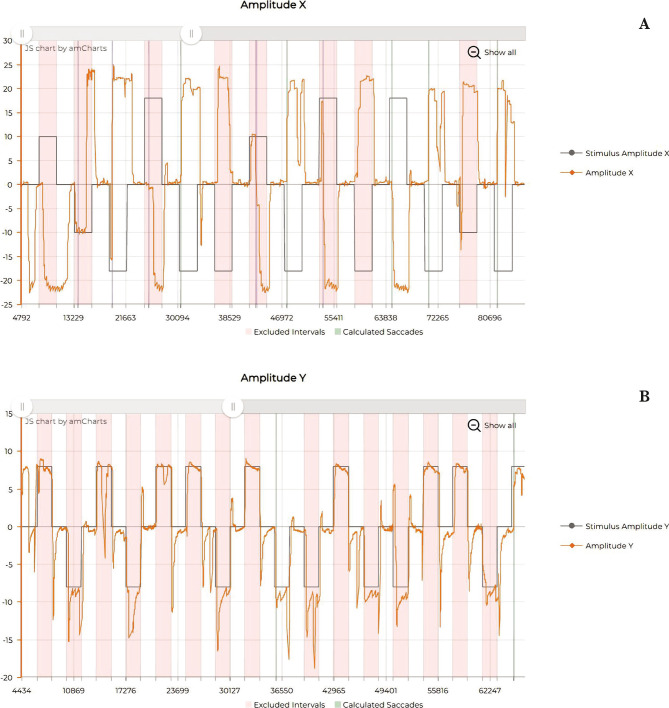
Eye tracking - Vertical saccades. A – Before rTMS treatment; B – After rTMS treatment.

**Figure 7:**
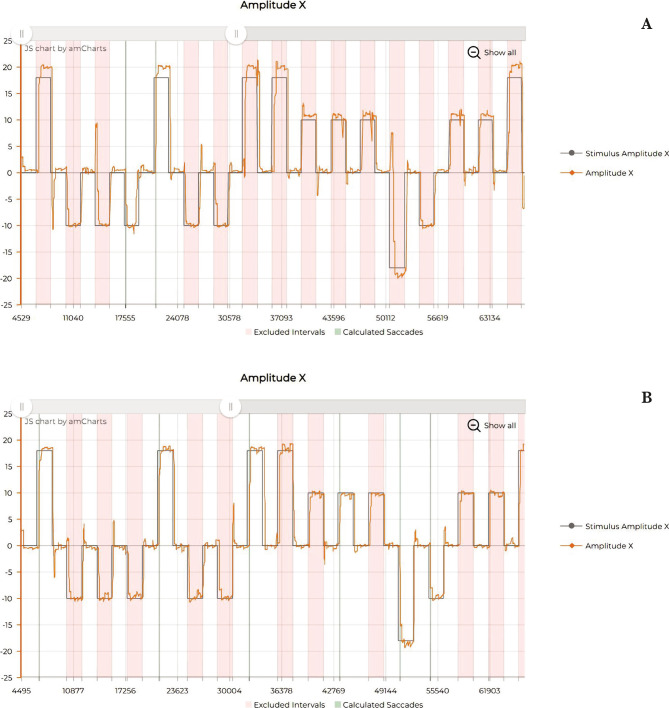
Eye tracking – Horizontal saccades. A – Before rTMS treatment; B – After rTMS treatment.

The evaluation through eye-tracking was also improved (Figure 5 A, B; 6 A, B and 7 A, B), showing a reduction of micro-saccadic flutter intrusions, the improvement of saccadic gain, and the uniformization of the mean velocity and peak velocity of the horizontal and vertical saccades.

After 6 months, we contacted the patient for medium to long-term follow-up evaluation. The patient suffered an oncologic relapse, thus being unable to present herself for the reevaluation.

## DISCUSSION

This case shows once more the importance of a complete evaluation of the patient with neurological symptoms. When the symptoms are not explained by any other etiology, it is mandatory to think about an onco-neuronal panel, as the clinical presentation of the PNS can be widely heterogenous [8]. The onco-neuronal antibodies seem to be linked with both the clinical syndrome and the type of cancer, anti-Yo antibodies being primarily found in PCA associated with gynecological tumors. Our patient also presented an important indicator of the high probability of neoplastic pathology: the presence of an unexplained neurological syndrome associated with a family history of neoplasms [2]. The presence of onco-neuronal antibodies is a marker of the presence of PNS and its autoimmune mechanism, but their pathophysiological role is still under debate [1].

PCA is a clinical syndrome, post-mortem studies showing that it is caused by the loss of Purkinje neurons. There are no specific paraclinical signs, except for the presence of onco-neuronal antibodies, the imagistic modification being mild and non-characteristic like a diffuse cerebellar atrophy on MRI [1].

rTMS is one of the non-invasive central nervous system stimulation techniques that have emerged in medical use in the last years, with the role of modulating brain activity and bringing new information about the cerebral pathways. One of the advantages of the rTMS intervention is its long-lasting effects on brain activity. Currently, the method is used as a therapeutic tool in many neurological and psychiatric pathologies. Recent studies showed that rTMS of the cerebellum could also be used for modulating the activity of different pathways, with long-term effects, such as the cerebello-thalamo-cortical (CTC), and limbic-thalamo-cortical networks, and subsequently influencing the excitability of areas that are connected functionally, although they are anatomically remote [9]. There are multiple case reports and case series showing a promising result of the inhibitory rTMS protocol of the posterior fossa in ataxia of different etiologies (genetic, inflammatory, degenerative etc) [5]. There is also a class II study, including 74 cases, that supports the benefits of this protocol in degenerative ataxia [4]. There is a need for additional studies to assess the benefits of rTMS therapy and establish the best protocol for patients with ataxia.

Several mechanisms that would explain the rTMS modulation effect on the cerebellum and the improvement of ataxia after cerebellar stimulation have been proposed, like oxidative stress reduction, cerebellar blood flow increase, or the effects that stimulation has on cerebellar-cortical plasticity [10]. One of these mechanisms is the CBI (cerebellar brain inhibition), an effect that has been observed when delivering a TMS pulse on the cerebellum, which will exert an inhibitory effect of the contralateral motor-cortex [11] through the CTC pathway controlled by inhibition from Purkinje cells [12]. Thus, the low-frequency stimulation will determine the suppression of the Purkinje cells, leading to the activation of the CTC pathway and disinhibition of the contralateral M1, improving the clinical features [13]. One of the ways proposed to increase the efficiency of the rTMS intervention is by using fMRI to establish the optimal stimulation area (a method that is superior to simply follow classical anatomical references) and the optimal stimulation protocol by identifying the excitability status of different areas and choosing the right action to undertake regarding different pathways [13].

This case report provides relevant insight into the role of rTMS intervention in ataxia. Further studies should be conducted to establish an optimal individualized treatment protocol, and other cases should be studied to assess the long-term efficiency of the procedure.
